# An elevated preoperative cholesterol-to-lymphocyte ratio predicts unfavourable outcomes in colorectal cancer liver metastasis patients receiving simultaneous resections: a retrospective study

**DOI:** 10.1186/s12893-023-01988-7

**Published:** 2023-05-16

**Authors:** Yiqiao Deng, Qichen Chen, Jinghua Chen, Yizhou Zhang, Jianjun Zhao, Xinyu Bi, Zhiyu Li, Yefan Zhang, Zhen Huang, Jianqiang Cai, Hong Zhao

**Affiliations:** Department of Hepatobiliary Surgery, National Clinical Research Center for Cancer/Cancer Hospital, National Cancer Center, Chinese Academy of Medical Sciences and Peking Union Medical College, Beijing, 100021 China

**Keywords:** Colorectal cancer liver metastases, Simultaneous resection, Cholesterol-to-lymphocyte ratio, Outcomes, Inverse probability of treatment weighting

## Abstract

**Background:**

To explore the clinical prognostic utility of the preoperative cholesterol-to-lymphocyte ratio (CLR) in outcomes for colorectal cancer liver metastasis (CRLM) patients receiving simultaneous resection of the primary lesion and liver metastases.

**Methods:**

A total of 444 CRLM patients receiving simultaneous resections were enrolled. The optimal cut-off value for CLR was determined using the highest Youden’s index. Patients were divided into the CLR < 3.06 group and the CLR≥3.06 group. Propensity score matching analysis (PSM) and the inverse probability of treatment weighting (IPTW) method were conducted to eliminate bias between the two groups. The outcomes included short-term outcomes and long-term outcomes. Kaplan–Meier curves and log-rank tests were used to analyse progression-free survival (PFS) and overall survival (OS).

**Results:**

In the short-term outcome analysis, after 1:1 PSM, 137 patients were distributed to the CLR < 3.06 group and CLR≥3.06 group. No significant difference was noted between the two groups (*P* > 0.1). Compared with patients with CLR < 3.06, patients with CLR≥3.06 had comparable operation times (320.0 [272.5–421.0] vs. 360.0 [292.5-434.5], *P =* 0.088), blood loss (200.0 [100.0-400.0] vs. 200.0 [150.0-450.0], *P* = 0.831), postoperative complication rates (50.4% vs. 46.7%, *P* = 0.546) and postoperative ICU rates (5.8% vs. 11.7%, *P =* 0.087). In the long-term outcome analysis, Kaplan–Meier analysis showed that compared with patients with CLR < 3.06, patients with CLR≥3.06 had worse PFS (*P* = 0.005, median: 10.2 months vs. 13.0 months) and OS (*P* = 0.002, median: 41.0 months vs. 70.9 months). IPTW-adjusted Kaplan–Meier analysis showed that the CLR≥3.06 group had worse PFS (*P* = 0.027) and OS (*P* = 0.010) than the CLR < 3.06 group. In the IPTW-adjusted Cox proportional hazards regression analysis, CLR≥3.06 was an independent factor for PFS (HR = 1.376, 95% CI 1.097–1.726, *P =* 0.006) and OS (HR = 1.723, 95% CI 1.218–2.439, *P* = 0.002). IPTW-adjusted Cox proportional hazards regression analysis including postoperative complications, operation time, intraoperative blood loss, intraoperative blood transfusion and postoperative chemotherapy revealed that CLR≥3.06 was an independent factor for PFS (HR = 1.617, 95% CI 1.252–2.090, *P <* 0.001) and OS (HR = 1.823, 95% CI 1.258–2.643, *P* = 0.002).

**Conclusions:**

The preoperative CLR level predicts unfavourable outcomes in CRLM patients receiving simultaneous resection of the primary lesion and liver metastases and should be taken into consideration when developing treatment and monitoring strategies.

**Supplementary Information:**

The online version contains supplementary material available at 10.1186/s12893-023-01988-7.

## Introduction

Colorectal cancer (CRC) is a digestive tract tumour, and liver metastases represent its most common malignant progression [1]. The 5-year survival rate of patients with colorectal cancer liver metastases (CRLM) is less than 10% [2] without surgical resection and approaches 50% [3] after active multimodal treatment comprising chemotherapy and hepatic resection. Surgical treatment remains the dominant contributor to the long-term survival of CRLM patients [4]. At colorectal cancer diagnosis, synchronous liver metastases occur in approximately 15% [2, 5] of patients. Simultaneous resection of primary lesions and liver metastases, an emerging curative resection for synchronous CRLM, has been increasingly [6] performed by surgeons. Compared with traditional staged resection, simultaneous resection can yield comparable short-term outcomes [6–9] and has a tendency to improve long-term outcomes [9]. Simultaneous resection is often associated with a shorter hospital stay, reduced hospital costs and a better experience for CRLM patients [7–9].

Due to the heterogeneous nature of tumours, the precise and personalized preoperative classification of surgery for CRLM patients could bring many benefits. There is a consensus [10, 11] that chronic inflammatory status instigates the initiation and development of cancer. Based on this notion, preoperative inflammatory biomarkers, such as the neutrophil-to-lymphocyte ratio (NLR) and the platelet-to-lymphocyte ratio (PLR), were established, and the utility of their prognostic value in multiple cancers was validated [12–14]. Among them, lymphocytes, especially tumour-infiltrating lymphocytes (TILs), played an essential role in the process of progression in CRC [15, 16]. More interestingly, peripheral lymphocyte counts were demonstrated to be useful in predicting the survival of triple-negative breast cancer [17], oral cancer [18] and colon cancer patients [19], and the prognostic utility of lymphocyte counts was reported to be better than that of other circulating serum cells, such as platelets and neutrophils [18].

Alteration of lipid metabolism is a hallmark in cancer. Cancer cells increase the uptake and storage of fatty acids, phospholipids and cholesterol, which supports the survival of cancer cells in a nutrient-poor microenvironment. These substances could also act as signalling molecules that activate tumour-related signalling pathways to promote proliferation, invasion and metastases [20–22]. Additionally, dysregulated cholesterol metabolism substantially promoted the progression of multiple cancers [23–25], including CRC [25]. The prognostic role of blood lipid markers, such as total cholesterol and high-density lipoprotein cholesterol (HDL-C), was reported in patients with endometrial cancer, non-small-cell lung cancer and CRC [26–28]. Recently, Zhou et al. [29] found that in CRC, the cholesterol-to-lymphocyte ratio (CLR), a marker that combines inflammatory status and lipid metabolism, was associated with long-term prognosis in CRC and exhibited more sensitivity and specificity than the common inflammatory marker NLR. However, the prognostic role of preoperative CLR in CRLM remains unknown. Given the abovementioned evidence, we aimed to verify and examine the predictive value of preoperative CLR in distinguishing the short-term and long-term prognosis of patients with CRLM who received simultaneous resection of the primary lesion and liver metastases.

## Methods

### Study population and variables

Collection and analysis of data in the study was performed after ethical approval (No. 81,972,311) was approved from the Institutional Review Board of the Cancer Hospital, Chinese Academy of Medical Sciences. The inclusion criteria were as follows: (1) histologically proven liver metastases of colorectal adenocarcinoma and (2) curative simultaneous resection of primary tumour and liver metastases. The exclusion criteria were as follows: (1) failure to follow-up or absence of clinical data; (2) other coexisting malignancies; and (3) presence of infectious disease before operation. In this study, we retrospectively collected the clinical information of 444 patients with CRLM who underwent simultaneous resection of primary lesions and liver metastases at the Cancer Hospital, Chinese Academy of Medical Sciences from January 2009 to November 2020.

Detailed information, including demographic characteristics (age, sex, body mass index (BMI), comorbidities, and American Society of Anaesthesiology (ASA) score), clinicopathological characteristics (preoperative cholesterol level, lymphocyte counts, neutrophil counts, and serum carcinoembryonic antigen (CEA)), tumour-related characteristics, treatment and oncological outcomes, was collected from every patient. In this study, comorbidity among CRLM patients was characterized as the presence of preoperative coexisting medical conditions or diseases, including but not limited to hypertension, diabetes, and dementia. All postoperative complications were evaluated using the Clavien-Dindo classification system. Complications were categorized as minor (Clavien-Dindo I-II) or major (Clavien-Dindo III-V) based on the severity of their impact on the patient’s health and recovery.

### Cholesterol-to-lymphocyte ratio (CLR)

Peripheral blood samples from each patient were collected within 1–3 weeks before the simultaneous resection. Preoperative CLR was defined by dividing total cholesterol by the lymphocyte count. The optimal cut-off value for CLR as a predictive tool for mortality in patients with CRLM undergoing simultaneous resection was determined using the highest Youden’s index (sensitivity + 1-specificity), which was graphically exhibited as the distance between the 45° line and the ROC. Then, patients were divided into two groups: patients with CLR < 3.06 in one group and patients with CLR≥3.06 in the other group.

### Treatment

The optimal treatment management protocol for CRLM patients was discussed and confirmed by a multidisciplinary team (MDT) composed of surgeons, oncologists and radiologists. The surgical data mainly included surgical margin (R0 resection or not), extent of liver resection (major resection or not), intraoperative radiofrequency ablation (RFA) and hepatic portal occlusion. Major resection was defined as resections of ≥ three segments of liver metastases. A combination of 5-fluorouracil/capecitabine and oxaliplatin/irinotecan with or without bevacizumab and cetuximab comprised the pretreatment chemotherapy regimen.

### Follow-up and outcomes

After surgery, patients were followed up with regular clinical examinations. The first postoperative follow-up was conducted one month from the date of surgery. Then, all follow-ups were regularly conducted every 3 months for 5 years and every 1 year thereafter.

The oncological outcomes were divided into short-term outcomes and long-term outcomes. The short-term outcomes included intraoperative operation time, intraoperative blood loss, postoperative hospital stay, incidence of postoperative complications and postoperative ICU rate. ICU rate is defined as the percentage of patients who required admission to the intensive care unit after surgery for any reason. The long-term outcomes included progression-free survival (PFS) and overall survival (OS). PFS was defined as the period of time from the date of surgical treatment to progression or the last follow-up date. OS was defined as the period of time from the date of surgical treatment to death or the last follow-up date.

### Statistical analysis

Continuous variables were measured as the median and interquartile range, and *t test*s or Mann–Whitney U tests were performed for comparison. Categorical variables were calculated as percentages and compared using the chi-square test. The preoperative NLR was calculated as (neutrophil count/lymphocyte count). The association of CLR with PFS and OS was firstly evaluated using univariable and multivariable Cox regression analyses. Only variables with *P* < 0.10 in the univariable analysis were included in the subsequent multivariable analysis. And the additional clinical net-benefit of CLR≥3.06 was assessed by employing decision curve analysis (DCA). To compare the short-term outcomes between the CLR < 3.06 group and CLR≥3.06 group, the propensity score matching (PSM) method was performed to balance the imbalanced covariates between the two groups. The inverse probability of treatment weighting (IPTW) method was performed to eliminate selection bias between the CLR < 3.06 group and the CLR≥3.06 group in the comparison of long-term outcomes.

The balance in covariates was evaluated using the standardized difference (SD) approach. A meaningful imbalance in the factors between the two groups was represented as an SD > 0.1. In the IPTW models, we retained all possible factors associated either with the CLR level or survival. We adopted adjusted Kaplan–Meier curves and log-rank tests to compare long-term outcomes (PFS and OS) between the CLR < 3.06 group and the CLR≥3.06 group. The inverse probability weighted Cox proportional hazards model was used to estimate the IPTW-adjusted hazard ratio (HR) of the level of CLR. *P* < 0.05 (two-sided) was considered to be statistically significant. Statistical analyses were performed using SPSS version 25 software (Armonk NY, USA) and R software (http://www.r-project.org).

## Results

### Clinicopathological characteristics

There were 444 patients enrolled in the present study with a median age of 59.0 (IQR [52.0, 65.0]) years. Greater than half of the patients were male (286/444, 64.4%), and comorbidities were present in 188 (42.3%) patients. The median NLR of the patients was 1.86 (IQR [1.34, 2.55]). Fifty-four (12.2%) patients had an ASA score of 3–4, whereas 209 (47.1%) patients had a BMI ≥ 24 kg/m^2^. Before simultaneous resection, 253 (57.0%) patients received pretreatment chemotherapy. For patients who had primary tumours located in the colon, the rate of patient population was 56.8%, and 89 (20.0%) among them had primary tumours located in the right hemicolon. For liver metastases, the median largest size was 2.5 cm (IQR [1.5, 4.0]), and the median number was 2 (IQR [1, 4]). Bilobular liver metastasis distribution was noted in 177 (39.9%) patients. Three hundred and twenty-one (72.3%) patients had positive lymph node metastasis. As depicted in the Figure S1, the optimal cutoff value of CLR for mortality was determined as 3.06. And two hundred eighty-five (64.2%) patients had CLR < 3.06, whereas 159 (35.8%) patients had CLR≥3.06. For patients who received all laparoscopic surgeries, the rate of the patient population was 21.8%. Concomitant RFA was performed in 43 (9.7%) patients, and the rate of hepatic portal occlusion among the patients was 69.6%. Two hundred and fourteen (48.2%) patients underwent major liver resection. The detailed clinicopathological characteristics of the patients are listed in Table 1.

**Table 1:** Clinicopathological characteristics and short-term outcomes in CRLM patients receiving simultaneous resection before PSM

Item	CLR < 3.06 (n = 285)	CLR ≥ 3.06 (n = 159)	*P*	All patients (n = 444)
Age ≥60 years, n (%)	126 (44.2%)	80 (50.3%)	0.216	206(46.4%)
Male, n (%)	185 (64.9%)	101 (63.5%)	0.769	286(64.4%)
BMI ≥ 24 kg/m^2^, n (%)	134 (47.0%)	75 (47.2%)	0.975	209(47.1%)
NLR ≥ 1.86, n (%)	113 (39.6%)	107 (67.3%)	< 0.001	220(49.5%)
Comorbidity, n (%)	123 (43.2%)	65 (40.9%)	0.641	188(42.3%)
ASA score 3–4, n (%)	30 (10.5%)	24 (15.1%)	0.158	54(12.2%)
Preoperative CEA ≥ 200 ng/ml, n (%)	10 ( 3.5%)	9 ( 5.7%)	0.283	19(4.3%)
Primary site in colon, n (%)	167 (58.6%)	85 (53.5%)	0.295	252(56.8%)
Right hemicolon, n (%)	53 (18.6%)	36 (22.6%)	0.307	89(20.0%)
Diameter of liver metastases ≥ 5 cm, n (%)	34 (11.9%)	29 (18.2%)	0.068	63(14.2%)
Multiple liver metastases, n (%)	168 (58.9%)	92 (57.9%)	0.824	260(58.6%)
Bilobar liver distribution, n (%)	108 (37.9%)	69 (43.4%)	0.256	177(39.9%)
Poor differentiation, n (%)	88 (30.9%)	56 (35.2%)	0.349	144(32.4%)
T3-T4 stage, n (%)	263 (92.3%)	146 (91.8%)	0.864	409(92.1%)
Positive lymph node metastasis, n (%)	206 (72.3%)	115 (72.3%)	0.992	321(72.3%)
Extrahepatic metastases, n (%)	30 (10.5%)	14 ( 8.8%)	0.561	44(9.9%)
Concomitant RFA, n (%)	25 ( 8.8%)	18 (11.3%)	0.384	43(9.7%)
R0 resection, n (%)	214 (75.1%)	119 (74.8%)	0.954	333(75.0%)
Major liver resection, n (%)	138 (48.4%)	76 (47.8%)	0.900	214(48.2%)
Pretreatment chemotherapy, n (%)	160 (56.1%)	93 (58.5%)	0.632	253(57.0%)
Hepatic portal occlusion, n (%)	192 (67.4%)	117 (73.6%)	0.172	309(69.6%)
All laparoscopic operation, n (%)	57 (20.0%)	40 (25.2%)	0.207	97(21.8%)
Operation time, min (median, IQR)	340.0(260.0-420.0)	320.0(270.0-430.0)	0.971	335.0(265.0-420.0)
Blood loss, ml (median, IQR)	200.0(100.0-400.0)	200.0(150.0-400.0)	0.163	200.0(100.0-400.0)
Blood transfusion, n (%)	66(23.2%)	37(23.3%)	0.979	103(23.2%)
Complications, n (%)				
0, n (%)	149(52.3%)	77(48.4%)	0.335	226(50.9%)
1–2, n (%)	83(29.1%)	43(27.0%)		126(28.4%)
3–5, n (%)	53(18.6%)	39(24.5%)		92(20.7%)
Postoperative minor complications, n (%)	83(29.1%)	43(27.0%)	0.641	126(28.4%)
Postoperative major complications, n (%)	53(18.6%)	39(24.5%)	0.139	92(20.7%)
ICU, n (%)	22(7.7%)	13(8.2%)	0.864	35(7.9%)
Post-operative hospital stay, days (median, IQR)	10.0(9.0–13.0)	10.0(8.0–14.0)	0.453	10.0(9.0–13.0)

### Short-term outcomes

The median operative time of patients was 335.0 min (IQR [265.0-420.0]). The median intraoperative blood loss volume was 200 ml (IQR [100, 400]), and 103 (23.2%) patients received an intraoperative blood transfusion. The median postoperative hospital stay duration was 10 days (IQR [9.0, 13.0]). Postoperative complications were observed in 218 (49.1%) patients, and 92 (20.7%) patients experienced major complications according to the Clavien-Dindo classification system. Thirty-five (7.9%) patients had postoperative ICU admission **(**Table 1**)**.

Compared to CLR < 3.06, patients with CLR≥3.06 had comparable operation times (320.0 [270.0-430.0] vs. 340.0 [260.0-420.0], *P =* 0.971), blood loss (200.0 [100.0-400.0] vs. 200.0 [150.0-400.0], *P* = 0.163), postoperative complication rates (51.6% vs. 47.7%, *P* = 0.436) and postoperative ICU rates (8.2% vs. 7.7%, *P =* 0.864).

**Fig. 1:**
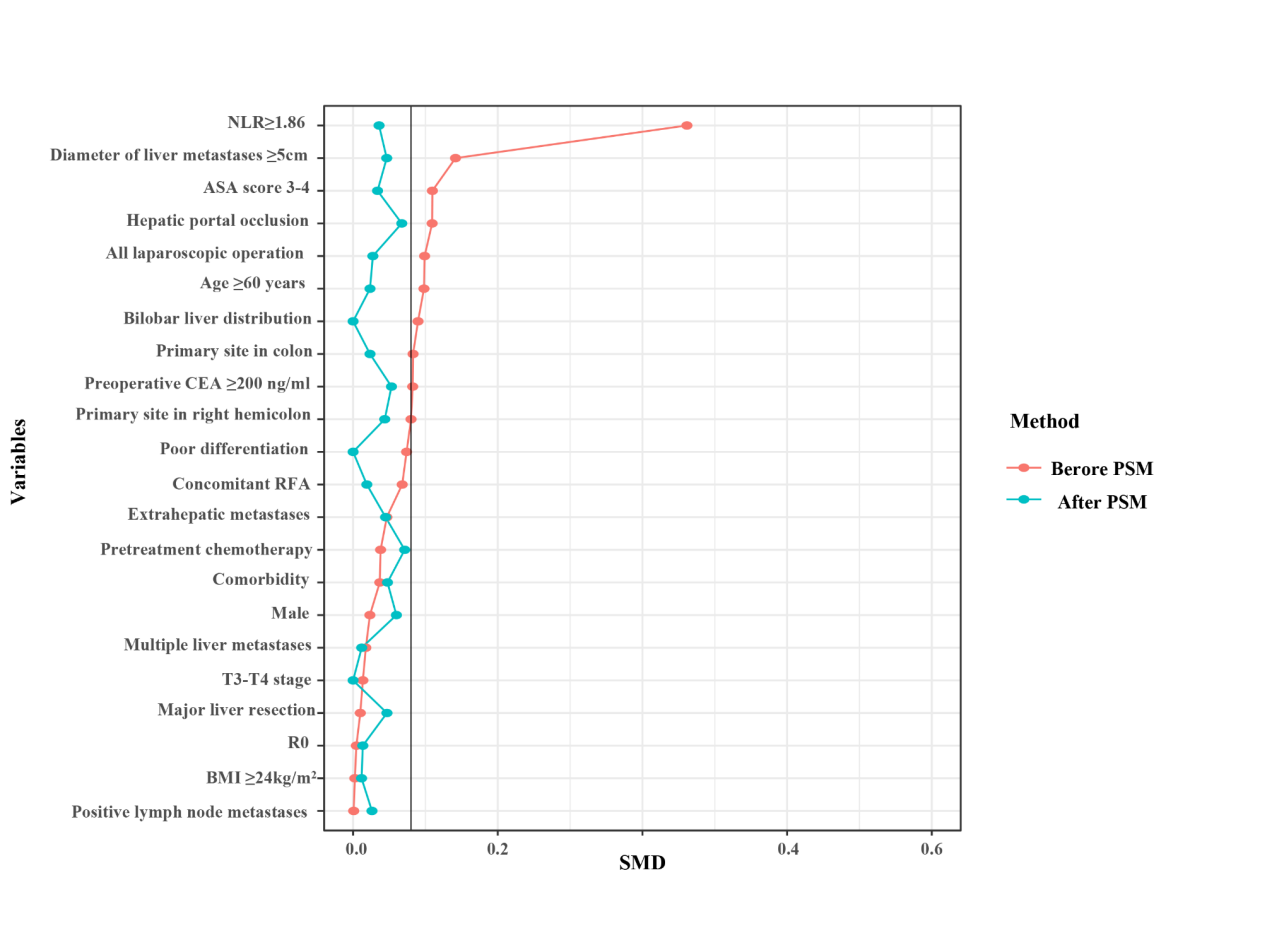
Standardized mean difference (SMD) of CLR < 3.06 vs. CLR ≥ 3.06 before and after PSM

After 1:1 PSM, 137 patients were distributed to the CLR < 3.06 group and CLR≥3.06 group **(**Fig. 1**).** No significant difference was noted between the two groups (*P* > 0.1). After PSM, compared with CLR < 3.06, patients with CLR≥3.06 had comparable operation times (320.0 [272.5–421.0] vs. 360.0 [292.5-434.5], *P =* 0.088), blood loss (200.0 [100.0-400.0] vs. 200.0 [150.0-450.0], *P* = 0.831), postoperative complication rates (50.4% vs. 46.7%, *P* = 0.546), and comparable postoperative ICU rates (5.8% vs. 11.7%, *P =* 0.087) **(**Table 2**)**.

**Table 2:** Clinicopathological characteristics and short-term outcomes in CRLM patients receiving simultaneous resection after PSM

Item	CLR < 3.06 (n = 137)	CLR ≥ 3.06 (n = 137)	*P*	All patients (n = 274)
Age ≥60 years, n (%)	65 (47.4%)	67 (48.9%)	0.809	132(48.2%)
Male, n (%)	81 (59.1%)	86 (62.8%)	0.536	167(60.9%)
BMI ≥ 24 kg/m^2^, n (%)	65 (47.4%)	64 (46.7%)	0.904	129(47.1%)
NLR ≥ 1.86, n (%)	82 (59.9%)	85 (62.0%)	0.710	167(60.9%)
Comorbidity, n (%)	58 (42.3%)	54 (39.4%)	0.623	112(40.9%)
ASA score 3–4, n (%)	18 (13.1%)	20 (14.6%)	0.727	38(13.9%)
Preoperative CEA ≥ 200 ng/ml, n (%)	6 ( 4.4%)	8 ( 5.8%)	0.583	14(5.1%)
Primary site in colon, n (%)	71 (51.8%)	73 (53.3%)	0.809	144(52.6%)
Right hemicolon, n (%)	26 (19.0%)	29 (21.2%)	0.651	55(20.1%)
Diameter of liver metastases ≥ 5 cm, n (%)	22 (16.1%)	25 (18.2%)	0.631	47(17.2%)
Multiple liver metastases, n (%)	82 (59.9%)	81 (59.1%)	0.902	163(59.5%)
Bilobar liver distribution, n (%)	57 (41.6%)	57 (41.6%)	1	114(41.6%)
Poor differentiation, n (%)	43 (31.4%)	43 (31.4%)	1	86(31.4%)
T3-T4 stage, n (%)	126 (92.0%)	126 (92.0%)	1	252(92.0%)
Positive lymph node metastasis, n (%)	98 (71.5%)	100 (73.0%)	0.787	198(72.3%)
Extrahepatic metastases, n (%)	9 ( 6.6%)	11 ( 8.0%)	0.642	20(7.3%)
Concomitant RFA, n (%)	15 (10.9%)	14 (10.2%)	0.844	29(10.6%)
R0 resection, n (%)	103 (75.2%)	102 (74.5%)	0.889	205(74.8%)
Major liver resection, n (%)	63 (46.0%)	67 (48.9%)	0.628	130(47.4%)
Pretreatment chemotherapy, n (%)	84 (61.3%)	78 (56.9%)	0.461	162(59.1%)
Hepatic portal occlusion, n (%)	105 (76.6%)	100 (73.0%)	0.486	205(74.8%)
All laparoscopic operation, n (%)	34 (24.8%)	32 (23.4%)	0.778	66(24.1%)
Operation time, min (median, IQR)	360.0(292.5-434.5)	320.0(272.5–421.0)	0.088	345.0(280.0-431.5)
Blood loss, ml (median, IQR)	200.0(150.0-450.0)	200.0(100.0-400.0)	0.831	200.0(100.0-400.0)
Blood transfusion, n (%%)	35(25.5%)	31(22.6%)	0.572	66(24.1%)
Complications, n (%)				
0, n (%)	73(53.5%)	68(49.6%)	0.814	141(51.5%)
1–2, n (%)	35(25.5%)	39(28.5%)		74(27.0%)
3–5, n (%)	29(21.2%)	30(21.9%)		59(21.5%)
Postoperative minor complications, n (%)	35(25.5%)	39(28.5%)	0.586	74(27.0%)
Postoperative major complications, n (%)	29(21.2%)	30(21.9%)	0.883	59(21.5%)
ICU, n (%)	16(11.7%)	8(5.8%)	0.087	24(8.8%)
Post-operative hospital stay, days (median, IQR)	10.0(9.0–13.0)	10.0(8.0–14.0)	0.913	10.0(9.0–14.0)

### Association with survival outcomes of CLR before IPTW adjustment

At the time of analysis, three hundred fifteen patients experienced recurrence, and 160 patients had died. The median PFS was 10.1 (IQR 4.1–23.2) months. The 1-year and 3-year PFS rates were 48.6% and 24.5%, respectively. The median OS was 30.9 (IQR 20.0-44.5) months. The 1-year, 3-year, and 5-year OS rates were 95.3%, 64.8% and 47.6%, respectively.

According to the univariable Cox regression analyses, CLR≥3.06 was found to be linked with PFS (Hazard ratio (HR) = 1.380, 95% CI 1.100–1.730; *P* = 0.006) and OS (HR = 1.630, 95% CI 1.190–2.230, *P* = 0.003). Furthermore, after adjusting for preoperative features in the multivariable Cox regression models, CLR≥3.06 continued to demonstrate a significant association with both PFS (HR = 1.400, 95% CI 1.110–1.780, *P* = 0.005) and OS (HR = 1.660, 95% CI 1.180–2.320, *P* = 0.003) (Table S2 & S3). Based on the DCA analysis results, CLR has the potential to produce more clinical net-benefit than several common clinical features in predicting both PFS and OS (Figure S2 & S3).

### Association with survival outcomes of CLR after IPTW adjustment

**Fig. 2:**
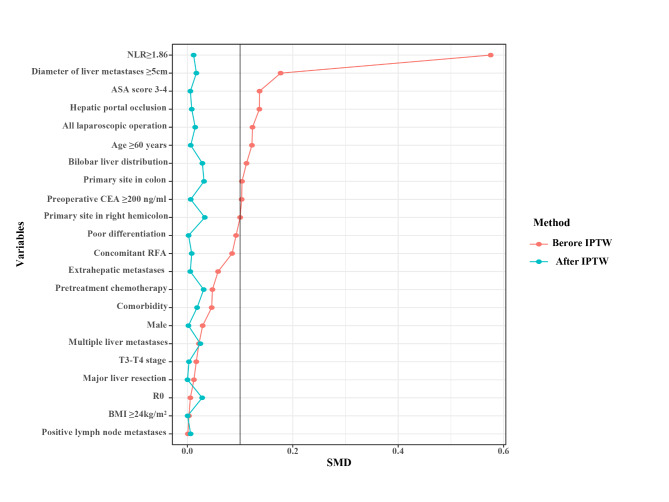
Standardized mean difference (SMD) of CLR < 3.06 vs. CLR ≥ 3.06 before and after IPTW

IPTW was performed to avoid bias between the CLR < 3.06 group and the CLR≥3.06 group. After IPTW adjustment, the SD for all characteristics was less than 0.1 **(**Fig. 2**)**, indicating that the weighted population was subsequently comparable.

**Fig. 3:**
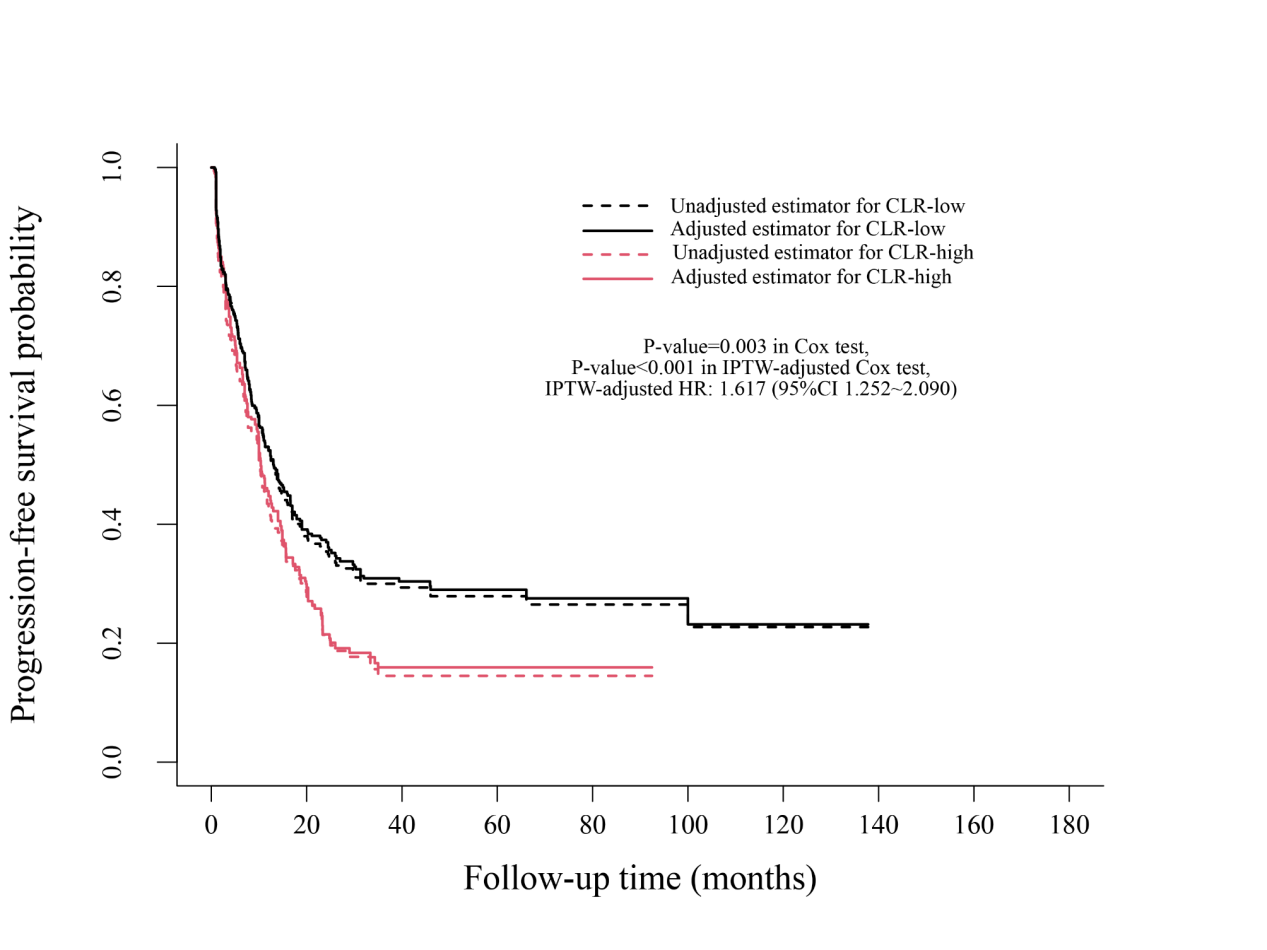
Progression-free survival analysis of CLR levels before and after IPTW

**Fig. 4:**
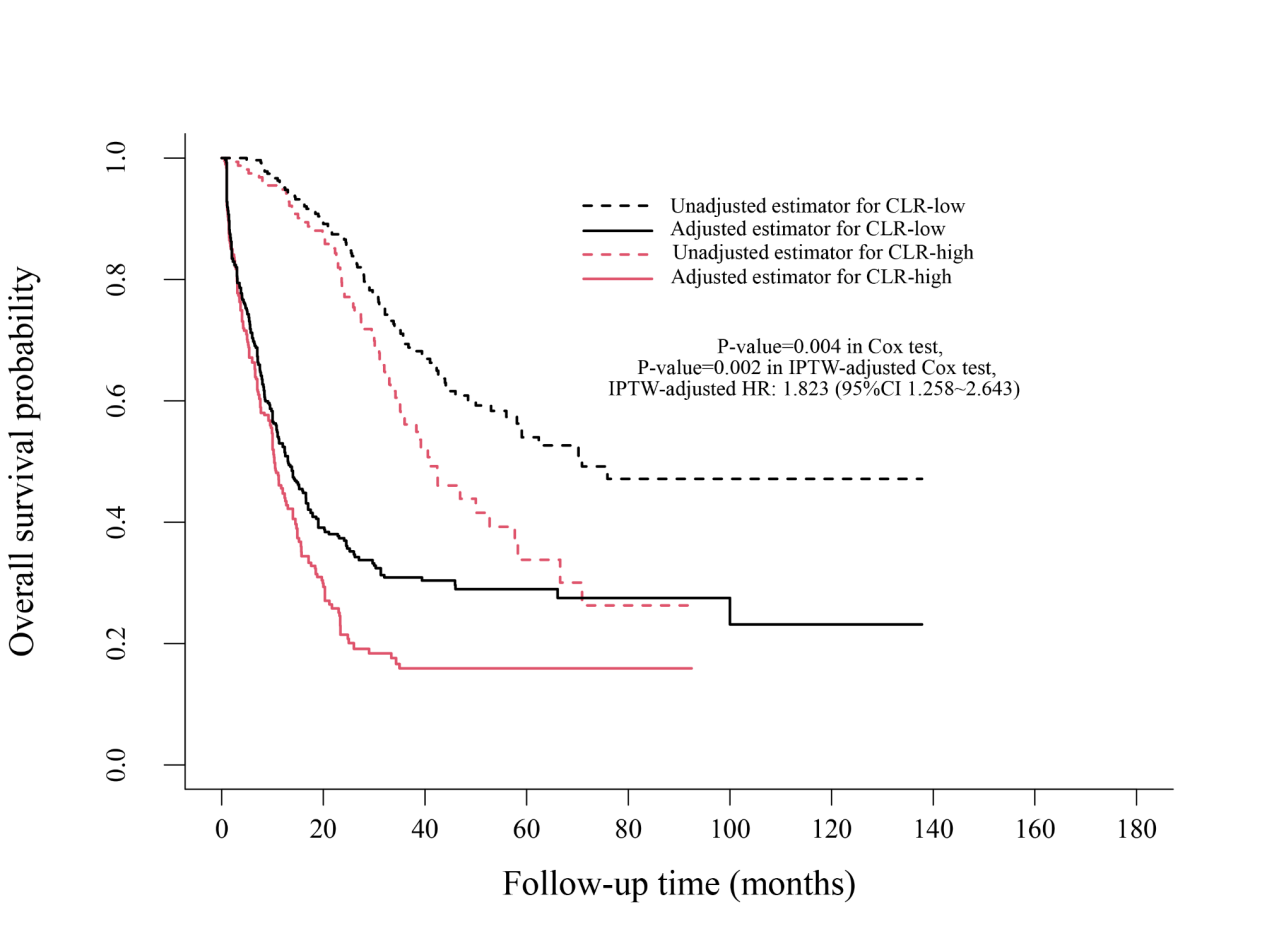
Overall survival analysis of CLR levels before and after IPTW

Kaplan–Meier analysis showed that compared with patients with CLR < 3.06, patients with CLR≥3.06 had a worse PFS (*P* = 0.005, median: 10.2 months vs. 13.0 months) and a worse OS (*P* = 0.002, median: 41.0 months vs. 70.9 months). IPTW-adjusted Kaplan–Meier analysis showed that patients with CLR≥3.06 had an unfavourable PFS (*P* = 0.027, median: 10.4 months vs. 13.1 months) and an unfavourable OS (*P* = 0.010, median: 42.5 months vs. 75.9 months) compared with those with CLR < 3.06 **(**Figs. 3 and 4**)**.

In the Cox proportional hazards regression analysis, CLR≥3.06 was significantly associated with worse PFS (HR = 1.376, 95% CI 1.097–1.726, *P =* 0.006) and OS (HR = 1.628, 95% CI 1.186–2.234, *P =* 0.003). In the IPTW-adjusted Cox proportional hazards regression analysis, CLR≥3.06 was an unfavourable risk factor for PFS (HR = 1.513, 95% CI 1.177–1.946, *P* = 0.001) and OS (HR = 1.723, 95% CI 1.218–2.439, *P* = 0.002).

When including postoperative complications, operation time, intraoperative blood loss and postoperative chemotherapy in the Cox proportional hazards regression analysis, CLR≥3.06 was still significantly associated with a worse PFS (HR = 1.415, 95% CI 1.123–1.782, *P* = 0.003) and a poor OS (HR = 1.611, 95% CI 1.166–2.226, *P =* 0.004). In the IPTW-adjusted Cox proportional hazards regression analysis, CLR≥3.06 was an unfavourable risk factor for PFS (HR = 1.617, 95% CI 1.252–2.090, *P <* 0.001) and OS (HR = 1.823, 95% CI 1.258–2.643, *P* = 0.002).

## Discussion

In this study, we first verified and rationalized the predictive effect of preoperative CLR on short-term and long-term prognosis in 444 patients with CRLM who received simultaneous resection of the primary lesion and liver metastases. PSM and IPTW were applied to eradicate the interfering bias between patients with CLR < 3.06 and patients with CLR ≥ 3.06. The main findings were as follows: (1) The preoperative CLR level could distinguish the long-term prognosis of patients with CRLM undergoing simultaneous resection of the primary lesion and liver metastases. Patients with CLR < 3.06 were associated with prolonged PFS and OS. (2) The preoperative CLR level could not distinguish the short-term prognosis of patients with CRLM undergoing simultaneous resection of the primary lesion and liver metastases. No differences in intraoperative operation time, intraoperative blood loss, postoperative hospital stay, incidence of postoperative complications or postoperative ICU rate were noted between the two groups.

Patients would benefit from convenient and reliable markers that could precisely identify their prognosis before receiving surgical resection or other treatment. In addition, this information would allow the surgeons perform more personalized management for patients since the allocation of medical resources would be more appropriate and satisfactory. To date, previous studies have mainly focused on patients with CRLM who receive staged resection, especially hepatic resection [30–32], and the subgroup of CRLM patients who receive simultaneous resection, as well as the specific surgical strategies employed (e.g., laparoscopic or open surgery) [33], have been overlooked. Interestingly, the prognostic effect of CLR in CRC was demonstrated in a recent study [29]; however, researchers only investigated the relationship between CLR and survival in these patients but ignored the possible association between CLR and short-term outcomes or recurrence. Nevertheless, the relatively smaller scale and lower disease stage of the 223 CRC patients compared to the 444 CRLM patients in our study also confined their efforts. This study is the first to holistically estimate the prognostic value of CLR on short- and long-term outcomes in CRLM patients who receive simultaneous resection of the primary lesion and liver metastases, contribute to accumulating evidence on the patient’s experience and provide new knowledge for the clinical guidance of this simultaneous resection procedure recommended by the NCCN guidelines [34] for surgeons. Given that the CLR level could be identified precisely and conveniently prior to surgery, this index would provide novel insight to optimize the risk stratification of CRLM patients.

In this study, we found that elevated CLR was associated with poor prognostic outcomes. Elevated CLR reflected elevated serum total cholesterol and/or decreased circulating lymphocyte counts. Inflammation plays a vital role in instigating the progression of multiple cancers, and a chronic inflammatory status could create a favourable tumour microenvironment (TME) to enable cancer cells to survive and proliferate, of which lymphocytes usually comprise an indispensable component. In general, lymphocytes include T cells, B cells and NK cells. These cells not only participate in host adaptive immunity but also reinforce innate immunity, thus being crucial to the antiviral and antitumour functions of cancer patients. The possible mechanisms explaining the finding that decreased lymphocyte counts were associated with poor prognosis are as follows. (1) Lymphocytes could regulate the secretion of potent cytotoxins, such as perforin [35], which contributed to both CD8 + and CD4 + CAR T-cell cytotoxicity and exerted the antitumour effect directly or indirectly. The depletion of lymphocyte count means weakened immune surveillance and antitumour immunity. (2) Decreased lymphocyte counts could be ascribed to the inflammation generated by tumours, and the inflammatory environment is always accompanied by DNA damage, impaired DNA repair function, degeneration of extracellular matrix, and a disrupted vascular barrier, all of which facilitate tumour progression [11, 36, 37].

Reprogrammed cholesterol metabolism was detected in renal carcinoma, breast cancer and CRC cells [23–25]. In this study, we found that when total cholesterol levels in serum increased, the increased CLR was also related to the poor prognosis of patients. It was reasonable to postulate the following possible mechanisms: (1) Elevated serum total cholesterol may reflect the overexpression of its rate-limiting enzyme squalene epoxidase (SQLE), which was demonstrated to be associated with the progression of CRC by activating CYP24A1-mediated MAPK signalling pathway [38]. In addition, it has also been reported that SQLE could promote CRC cells to resist apoptosis and proliferate by triggering gut microbiota dysbiosis and impairing gut barrier function [39]. It is worth mentioning that the accumulation of cholesterol was found to be able to conversely reduce SQLE, which triggered the chain reaction, including activation of β-catenin and inactivation of the p53 tumour-suppressive pathway, thereby aggravating CRC progression [40]. (2) In addition, previous research also reported that the SREBP2-dependent cholesterol biosynthesis pathway was exclusively activated in the liver metastases of CRC [41] but not metastases to the brain or lung. SREBP2 could help circulating tumour cells to resist ferroptosis and acquire drug resistance by upregulating transcription of the iron carrier Transferrin (TF) [42]. The interaction between cholesterol and lymphocytes may also have an important impact on cancer progression. Cholesterol could cause alterations in intestinal immunity and impair intestinal innate immunity by restraining the differentiation of IgA plasma cells [43]. In addition, it has been suggested that cholesterol might disturb the normal function of activated T cells, such as CD8^+^ T cells, which play an important role in antitumor immunity by regulating immune checkpoint expression levels [44] or specifically combining with the TCR-CD3 complex and directly stopping TCR signalling in T cells [45]. It was also important to emphasize the fact that long-term dietary and behavioural habits are the main reasons explaining changes in the serum cholesterol level [46]. Acute aberrations in serum cholesterol levels before surgery were rarely observed based on our clinical experience. Therefore, the stability and reliability of the index were assured.

Although statistical methods, such as PSM and IPTW, were both applied to eliminate confounding interference between patients in the CLR < 3.06 group and patients in the CLR ≥ 3.06 group, inevitable innate bias could not be completely avoided due to the retrospective nature of the study. In addition, although up to 444 CRLM patients were enrolled in this study, the study size was still relatively small. The single-centre design also constrained our efforts and potentially affected the representativeness of our research. And lack of gene mutation data is also a constraint of our study, we will perform large-scale, multicentre and prospective studies to further verify our findings in the future.

## Conclusions

In conclusion, we combined the influence of inflammation and lipid metabolism to validate the predictive value of preoperative CLR on the short- and long-term prognosis of 444 CRLM patients who received simultaneous resection. These findings provide novel insights into the characteristics of CRLM patients and conferred new knowledge to surgeons to guide the management of CRLM patients.

## Electronic supplementary material

Below is the link to the electronic supplementary material.

Additional File 1: STARD-Checklist

Additional File 2: Supplementary Tables

Additional File 3: Supplementary Figures

## Data Availability

The datasets analysed during the current study are available from the corresponding author (H. Z., Email: pumc95zhao@126.com) on reasonable request.

## Abbreviations

CLR: Cholesterol-to-lymphocyte ratio
CRLM: Colorectal cancer liver metastasis
IPTW: Inverse probability of treatment weighting
PFS: Progression-free survival
OS: Overall survival
CRC: Colorectal cancer
TILs: Tumour-infiltrating lymphocytes
HDL-C: High-density lipoprotein cholesterol
BMI: Body mass index
ASA: American Society of Anaesthesiology
CEA: Carcinoembryonic antigen
MDT: Multidisciplinary team
RFA: Radiofrequency ablation
DCA: Decision curve analysis
NLR: Neutrophil-to-lymphocyte ratio
SD: Standardized difference
HR: Hazard ratio

## References

[1] Adam R (2003). Chemotherapy and surgery: new perspectives on the treatment of unresectable liver metastases. Ann Oncol.

[2] Manfredi S, Lepage C, Hatem C, Coatmeur O, Faivre J, Bouvier AM. Epidemiology and management of liver metastases from colorectal cancer. Ann Surg. 2006 Aug;244(2):254–9. 10.1097/01.sla.0000217629.94941.cf. 10.1097/01.sla.0000217629.94941.cf PMC1602156 16858188

[3] Imai K, Benitez CC, Allard MA, et al. Impact of Surgical Treatment for Recurrence after 2-Stage Hepatectomy for Colorectal Liver Metastases, on patient outcome. Ann Surg. 2019 Feb;269(2):322–30. 10.1097/SLA.0000000000002472. 10.1097/SLA.0000000000002472 28820745

[4] Datta J, Narayan RR, Kemeny NE, D’Angelica MI. Role of Hepatic Artery Infusion Chemotherapy in Treatment of Initially Unresectable Colorectal Liver Metastases: A Review. JAMA Surg. 2019 Aug 1;154(8):768–776. 10.1001/jamasurg.2019.1694. 10.1001/jamasurg.2019.1694 31188415

[5] Engstrand J, Nilsson H, Strömberg C (2018). Colorectal cancer liver metastases - a population-based study on incidence, management and survival. BMC Cancer.

[6] Wang LJ, Wang HW, Jin KM, Li J, Xing BC. Comparison of sequential, delayed and simultaneous resection strategies for synchronous colorectal liver metastases.BMC Surg. 2020 Jan17;20(1):16. 10.1186/s12893-020-0681-7. 10.1186/s12893-020-0681-7 PMC6969459 31952490

[7] Ejaz A, Semenov E, Spolverato G, et al. Synchronous primary colorectal and liver metastasis: impact of operative approach on clinical outcomes and hospital charges. HPB (Oxford). 2014 Dec;16(12):1117–26. 10.1111/hpb.12302. Epub 2014 Jun 26. 10.1111/hpb.12302 PMC4253336 24965845

[8] Dulundu E, Attaallah W, Tilki M, Yegen C et al. Simultaneous resection for CRC with synchronous liver metastases is a safe procedure: Outcomes at a single center in Turkey. Biosci Trends. 2017 May 23;11(2):235–242. 10.5582/bst.2017.01019. 10.5582/bst.2017.01019 28216517

[9] Boudjema K, Locher C, Sabbagh C et al. Simultaneous Versus Delayed Resection for Initially Resectable Synchronous CRC Liver Metastases: A Prospective, Open-label, Randomized, Controlled Trial. Ann Surg. 2021 Jan 1;273(1):49–56. 10.1097/SLA.0000000000003848. 10.1097/SLA.0000000000003848 32209911

[10] Elinav E, Nowarski R, Thaiss CA, Hu B, Jin C, Flavell RA. Inflammation-induced cancer: crosstalk between tumours, immune cells and microorganisms. Nat Rev Cancer. 2013 Nov;13(11):759–71. 10.1038/nrc3611. 10.1038/nrc3611 24154716

[11] Diakos CI, Charles KA, McMillan DC, Clarke SJ. Cancer-related inflammation and treatment effectiveness. Lancet Oncol. 2014 Oct;15(11):e493–503. 10.1016/S1470-2045(14)70263-3. 10.1016/S1470-2045(14)70263-3 25281468

[12] Koh YW, Choi JH, Ahn MS, Choi YW, Lee HW. Baseline neutrophil-lymphocyte ratio is associated with baseline and subsequent presence of brain metastases in advanced non-small-cell lung cancer.Sci Rep. 2016 Dec7;6:38585. 10.1038/srep38585. 10.1038/srep38585 PMC5141478 27924837

[13] Fang T, Wang Y, Yin X et al. Diagnostic Sensitivity of NLR and PLR in Early Diagnosis of Gastric Cancer.J Immunol Res. 2020 Mar7;2020:9146042. 10.1155/2020/9146042. 10.1155/2020/9146042 PMC7081040 32211444

[14] Ding N, Pang Z, Shen H, Ni Y, Du J, Liu Q. The Prognostic Value of PLR in Lung Cancer, a Meta-analysis Based on Results from a Large Consecutive Cohort.Sci Rep. 2016 Oct5;6:34823. 10.1038/srep34823. 10.1038/srep34823 PMC5050506 27703265

[15] Bai Z, Zhou Y, Ye Z, Xiong J, Lan H, Wang F. tumour-Infiltrating Lymphocytes in CRC: The Fundamental Indication and Application on Immunotherapy.Front Immunol. 2022 Jan14;12:808964. 10.3389/fimmu.2021.808964. 10.3389/fimmu.2021.808964 PMC8795622 35095898

[16] Wang S, Qu Y, Xia P, et al. Transdifferentiation of tumour infiltrating innate lymphoid cells during progression of CRC. Cell Res. 2020 Jul;30(7):610–22. 10.1038/s41422-020-0312-y. 10.1038/s41422-020-0312-y PMC7343789 32367039

[17] Afghahi A, Purington N, Han SS et al. Higher Absolute lymphocyte count Predict Lower Mortality from Early-Stage Triple-Negative Breast Cancer.Clin Cancer Res. 2018 Jun15;24(12):2851–2858. 10.1158/1078-0432.CCR-17-1323. 10.1158/1078-0432.CCR-17-1323 PMC6366842 29581131

[18] Diao P, Wu Y, Ge H et al. Preoperative circulating platelet, neutrophil, and lymphocyte count predict survival in oral cancer.Oral Dis. 2019May;25(4):1057–1066. 10.1111/odi.13049. 10.1111/odi.13049 30697882

[19] Noh OK, Oh SY, Kim YB, Suh KW. Prognostic significance of lymphocyte count in Colon cancer patients treated with FOLFOX Chemotherapy. World J Surg. 2017 Nov;41(11):2898–905. 10.1007/s00268-017-4104-6. 10.1007/s00268-017-4104-6 28707088

[20] Röhrig F, Schulze A. The multifaceted roles of fatty acid synthesis in cancer. Nat Rev Cancer. 2016 Nov;16(11):732–49. 10.1038/nrc.2016.89. 10.1038/nrc.2016.89 27658529

[21] Cheng C, Geng F, Cheng X, Guo D. Lipid metabolism reprogramming and its potential targets in cancer. Cancer Commun (Lond). 2018 May 21;38(1):27. 10.1186/s40880-018-0301-4. 10.1186/s40880-018-0301-4 PMC5993136 29784041

[22] Cao Y. Adipocyte and lipid metabolism in cancer drug resistance. J Clin Invest. 2019 Jul 2;129(8):3006–3017. 10.1172/JCI127201. 10.1172/JCI127201 PMC6668696 31264969

[23] Liu Z, Liu X, Liu S, Cao Q. Cholesterol promotes the migration and invasion of renal carcinoma cells by regulating the KLF5/miR-27a/FBXW7 pathway.Biochem Biophys Res Commun. 2018 Jul7;502(1):69–75. 10.1016/j.bbrc.2018.05.122. 10.1016/j.bbrc.2018.05.122 29782853

[24] Voisin M, de Medina P, Mallinger A et al. Identification of a tumour-promoter cholesterol metabolite in human breast cancers acting through the glucocorticoid receptor. Proc Natl Acad Sci U S A. 2017 Oct 31;114(44):E9346-E9355. 10.1073/pnas.1707965114. 10.1073/pnas.1707965114 PMC5676900 29078321

[25] Wang Y, Liu C, Hu L. Cholesterol regulates cell proliferation and apoptosis of CRC by modulating miR-33a-PIM3 pathway. Biochem Biophys Res Commun. 2019 Apr 9;511(3):685–692. 10.1016/j.bbrc.2019.02.123. 10.1016/j.bbrc.2019.02.123 30827510

[26] Luo YZ, Yang Z, Qiu YL et al. Pretreatment triglycerides-to-high density lipoprotein cholesterol ratio in postmenopausal women with endometrial cancer.Kaohsiung J Med Sci. 2019May;35(5):303–309. 10.1002/kjm2.12033. 10.1002/kjm2.12033 PMC11900726 30887645

[27] Luo F, Zeng KM, Cao JX et al. Predictive value of a reduction in the level of high-density lipoprotein-cholesterol in patients with non-small-cell lung cancer undergoing radical resection and adjuvant chemotherapy: a retrospective observational study.Lipids Health Dis. 2021 Sep20;20(1):109. 10.1186/s12944-021-01538-1. 10.1186/s12944-021-01538-1 PMC8454045 34544437

[28] Zhou P, Li B, Liu B, Chen T, Xiao J. Prognostic role of serum total cholesterol and high-density lipoprotein cholesterol in cancer survivors: A systematic review and meta-analysis.Clin Chim Acta. 2018 Feb;477:94–104. 10.1016/j.cca.2017.11.039. 10.1016/j.cca.2017.11.039 29223765

[29] Zhou S, He Q, Sheng N, Gong J, Ren J, Wang Z. Blood cholesterol-to-lymphocyte ratio as a novel prognostic marker to predict postoperative overall survival in patients with CRC.World J Surg Oncol. 2022 Jan15;20(1):18. 10.1186/s12957-021-02471-4. 10.1186/s12957-021-02471-4 PMC8760814 35033097

[30] Halazun KJ, Aldoori A, Malik HZ et al. Elevated preoperative neutrophil to lymphocyte ratio predicts survival following hepatic resection for colorectal liver metastases.Eur J Surg Oncol. 2008Jan;34(1):55–60. 10.1016/j.ejso.2007.02.014. 10.1016/j.ejso.2007.02.014 17448623

[31] Giakoustidis A, Neofytou K, Khan AZ, Mudan S. Neutrophil to lymphocyte ratio predicts pattern of recurrence in patients undergoing liver resection for colorectal liver metastasis and thus the overall survival. J Surg Oncol. 2015 Mar 15;111(4):445 – 50. 10.1002/jso.23845. 10.1002/jso.23845 25557840

[32] Cimino MM, Donadon M, Giudici S et al. Peri-tumoural CD3 + Inflammation and Neutrophil-to-Lymphocyte Ratio Predict Overall Survival in Patients Affected by Colorectal Liver Metastases Treated with Surgery.J Gastrointest Surg. 2020May;24(5):1061–1070. 10.1007/s11605-019-04458-9. 10.1007/s11605-019-04458-9 31823322

[33] Sena G, Picciariello A, Marino F et al. One-Stage Total Laparoscopic Treatment for Colorectal Cancer With Synchronous Metastasis. Is It Safe and Feasible? Front Surg.2021 Nov18;8:752135. 10.3389/fsurg.2021.752135. 10.3389/fsurg.2021.752135 PMC8637405 34869559

[34] Benson AB, Venook AP, Al-Hawary MM et al. Colon Cancer, Version 2.2021, NCCN Clinical Practice Guidelines in Oncology. J Natl Compr Canc Netw. 2021 Mar 2;19(3):329–359. 10.6004/jnccn.2021.0012. 10.6004/jnccn.2021.0012 33724754

[35] Ishii K, Pouzolles M, Chien CD et al. Perforin-deficient CAR T cells recapitulate late-onset inflammatory toxicities observed in patients. J Clin Invest. 2020 Oct 1;130(10):5425–5443. 10.1172/JCI130059. 10.1172/JCI130059 PMC7524496 32925169

[36] Kay J, Thadhani E, Samson L, Engelward B. Inflammation-induced DNA damage, mutations and cancer. DNA Repair (Amst). 2019 Nov;83:102673. 10.1016/j.dnarep.2019.102673. Epub 2019 Jul 25. 10.1016/j.dnarep.2019.102673 PMC6801086 31387777

[37] Kidane D, Chae WJ, Czochor J, et al. Interplay between DNA repair and inflammation, and the link to cancer. Crit Rev Biochem Mol Biol. 2014 Mar-Apr;49(2):116–39. 10.3109/10409238.2013.875514. 10.3109/10409238.2013.875514 PMC4300235 24410153

[38] He L, Li H, Pan C, et al. Squalene epoxidase promotes CRC cell proliferation through accumulating calcitriol and activating CYP24A1-mediated MAPK signaling. Cancer Commun (Lond). 2021 Aug;41(8):726–46. 10.1002/cac2.12187. 10.1002/cac2.12187 PMC8360641 34268906

[39] Li C, Wang Y, Liu D et al. Squalene epoxidase drives cancer cell proliferation and promotes gut dysbiosis to accelerate colorectal carcinogenesis. Gut. 2022 Mar 1:gutjnl-2021-325851. 10.1136/gutjnl-2021-325851. 10.1136/gutjnl-2021-325851 PMC9554078 35232776

[40] Jun SY, Brown AJ, Chua NK, et al. Reduction of Squalene epoxidase by cholesterol Accumulation accelerates CRC Progression and Metastasis. Gastroenterology. 2021 Mar;160(4):1194–1207e28. 10.1053/j.gastro.2020.09.009. 10.1053/j.gastro.2020.09.009 32946903

[41] Zhang KL, Zhu WW, Wang SH et al. Organ-specific cholesterol metabolic aberration fuels liver metastasis of CRC.Theranostics. 2021 Apr27;11(13):6560–6572. 10.7150/thno.55609. 10.7150/thno.55609 PMC8120208 33995676

[42] Hong X, Roh W, Sullivan RJ, et al. The Lipogenic Regulator SREBP2 induces transferrin in circulating Melanoma cells and suppresses ferroptosis. Cancer Discov. 2021 Mar;11(3):678–95. 10.1158/2159-8290.CD-19-1500. 10.1158/2159-8290.CD-19-1500 PMC7933049 33203734

[43] Trindade BC, Ceglia S, Berthelette A et al. The cholesterol metabolite 25-hydroxycholesterol restrains the transcriptional regulator SREBP2 and limits intestinal IgA plasma cell differentiation. Immunity. 2021 Oct 12;54(10):2273–2287.e6. 10.1016/j.immuni.2021.09.004. 10.1016/j.immuni.2021.09.004 PMC8570345 34644558

[44] Ma X, Bi E, Lu Y et al. Cholesterol Induces CD8 + T Cell Exhaustion in the tumour Microenvironment. Cell Metab. 2019 Jul 2;30(1):143–156.e5. 10.1016/j.cmet.2019.04.002. 10.1016/j.cmet.2019.04.002 PMC7061417 31031094

[45] Chen Y, Zhu Y, Li X et al. Cholesterol inhibits TCR signaling by directly restricting TCR-CD3 core tunnel motility. Mol Cell. 2022 Mar 3:S1097-2765(22)00155-1. 10.1016/j.molcel.2022.02.017. 10.1016/j.molcel.2022.02.017 35271814

[46] NCD Risk Factor Collaboration (NCD-RisC). Repositioning of the global epicentre of non-optimal cholesterol.Nature. 2020 Jun;582(7810):73–77. 10.1038/s41586-020-2338-1. 10.1038/s41586-020-2338-1 PMC7332422 32494083

