# Supplementary material for: An elevated preoperative cholesterol-to-lymphocyte ratio predicts unfavourable outcomes in colorectal cancer liver metastasis patients receiving simultaneous resections: a retrospective study
Source: BMC Surg. 2023 May 16;23:131. doi: 10.1186/s12893-023-01988-7 (PMC10190004; doi:10.1186/s12893-023-01988-7)
Supplement: Supplementary file 2 — Additional File 2: Supplementary Tables [file 12893_2023_1988_MOESM2_ESM.docx]

**Table S1. Selected baseline characteristics**

**between CLR<3.06 and CLR≥3.06 before and after weighting**

| **Factor** | **Unweighted Study Population, No. (%)** | | | **Weighted Study Population, %** | | |
| --- | --- | --- | --- | --- | --- | --- |
|  | **CLR≥3.06**  **(n=159)** | **CLR<3.06**  **(n=285)** | **Standardized difference** | **CLR≥3.06**  **(n=137)** | **CLR<3.06**  **(n=137)** | **Standardized difference** |
| Age ≥60 years, n (%) | 126 (44.2%) | 80 (50.3%) | 0.122 | 65 (47.4%) | 67 (48.9%) | 0.029 |
| Male, n (%) | 185 (64.9%) | 101 (63.5%) | 0.029 | 81 (59.1%) | 86 (62.8%) | 0.075 |
| BMI≥24kg/m^2^, n (%) | 134 (47.0%) | 75 (47.2%) | 0.003 | 65 (47.4%) | 64 (46.7%) | 0.015 |
| Comorbidity, n (%) | 113 (39.6%) | 107 (67.3%) | 0.046 | 82 (59.9%) | 85 (62.0%) | 0.059 |
| NLR≥1.86, n(%) | 123 (43.2%) | 65 (40.9%) | 0.577 | 58 (42.3%) | 54 (39.4%) | 0.045 |
| ASA score 3-4, n (%) | 30 (10.5%) | 24 (15.1%) | 0.137 | 18 (13.1%) | 20 (14.6%) | 0.042 |
| Preoperative CEA ≥200 ng/ml, n (%) | 10 ( 3.5%) | 9 ( 5.7%) | 0.103 | 6 ( 4.4%) | 8 ( 5.8%) | 0.066 |
| Primary site in colon, n (%) | 167 (58.6%) | 85 (53.5%) | 0.104 | 71 (51.8%) | 73 (53.3%) | 0.029 |
| Right hemicolon, n (%) | 53 (18.6%) | 36 (22.6%) | 0.100 | 26 (19.0%) | 29 (21.2%) | 0.055 |
| Diameter of liver metastases ≥5 cm, n (%) | 34 (11.9%) | 29 (18.2%) | 0.177 | 22 (16.1%) | 25 (18.2%) | 0.058 |
| Multiple liver metastases, n (%) | 168 (58.9%) | 92 (57.9%) | 0.022 | 82 (59.9%) | 81 (59.1%) | 0.015 |
| Bilobar liver distribution, n (%) | 108 (37.9%) | 69 (43.4%) | 0.112 | 57 (41.6%) | 57 (41.6%) | <0.001 |
| Poor differentiation, n (%) | 88 (30.9%) | 56 (35.2%) | 0.092 | 43 (31.4%) | 43 (31.4%) | <0.001 |
| T3-T4 stage, n (%) | 263 (92.3%) | 146 (91.8%) | 0.017 | 126 (92.0%) | 126 (92.0%) | <0.001 |
| Positive lymph node metastasis, n (%) | 206 (72.3%) | 115 (72.3%) | 0.001 | 98 (71.5%) | 100 (73.0%) | 0.033 |
| Extrahepatic metastases, n (%) | 30 (10.5%) | 14 ( 8.8%) | 0.058 | 9 ( 6.6%) | 11 ( 8.0%) | 0.056 |
| Concomitant RFA, n (%) | 25 ( 8.8%) | 18 (11.3%) | 0.085 | 15 (10.9%) | 14 (10.2%) | 0.024 |
| R0 resection, n (%) | 214 (75.1%) | 119 (74.8%) | 0.006 | 103 (75.2%) | 102 (74.5%) | 0.017 |
| Major liver resection, n (%) | 138 (48.4%) | 76 (47.8%) | 0.012 | 63 (46.0%) | 67 (48.9%) | 0.058 |
| Pretreatment chemotherapy, n (%) | 160 (56.1%) | 93 (58.5%) | 0.048 | 84 (61.3%) | 78 (56.9%) | 0.089 |
| Hepatic portal occlusion, n (%) | 192 (67.4%) | 117 (73.6%) | 0.137 | 105 (76.6%) | 100 (73.0%) | 0.084 |
| All laparoscopic operation, n (%) | 57 (20.0%) | 40 (25.2%) | 0.124 | 34 (24.8%) | 32 (23.4%) | 0.034 |

**Table S2. Prognostic factors for PFS in CRLM patients**

| **Factor** | **Univariate analysis** | | **Multivariate analysis** | |
| --- | --- | --- | --- | --- |
|  | ***P*** | **HR (95%CI)** | ***P*** | **HR (95%CI)** |
| Age≥60 years | 0.586 | 0.940 (0.753-1.170) |  |  |
| Male | 0.303 | 1.130 (0.896-1.420) |  |  |
| BMI≥24kg/m^2^ | 0.266 | 1.130 (0.909-1.410) |  |  |
| Comorbidity | 0.905 | 1.010 (0.811-1.270) |  |  |
| ASA score 3-4 | 0.725 | 0.941 (0.670-1.320) |  |  |
| CEA≥200 ng/ml | <0.001 | 1.380 (0.846-2.250) |  |  |
| Primary site in colon | 0.773 | 0.968 (0.774-1.210) |  |  |
| Right hemicolon | 0.850 | 0.973 (0.735-1.290) |  |  |
| Diameter of liver metastases≥5 cm | 0.043 | 1.430 (1.010-2.010) |  |  |
| Multiple liver metastases | <0.001 | 1.710 (1.350-2.150) |  |  |
| Bilobar liver distribution | <0.001 | 1.730 (1.380-2.160) |  |  |
| Poor differentiation | 0.095 | 1.220 (0.966-1.550) |  |  |
| T3-T4 stage | <0.001 | 1.450 (0.924-2.290) |  |  |
| Positive lymph node metastasis | <0.001 | 2.060 (1.560-2.700) | <0.001 | 1.890 (1.420-2.500) |
| Extrahepatic metastases | <0.001 | 2.140 (1.520-3.000) | <0.001 | 1.870 (1.320-2.660) |
| Concomitant RFA | <0.001 | 1.840 (1.310-2.590) |  |  |
| R0 resection | <0.001 | 0.585 (0.458-0.747) | 0.007 | 0.703 (0.543-0.910) |
| Major liver resection | <0.001 | 1.820 (1.460-2.280) | 0.038 | 1.380 (1.020-1.880) |
| Hepatic portal occlusion | 0.146 | 1.200 (0.940-1.520) |  |  |
| All laparoscopic operation | <0.001 | 0.652 (0.486-0.874) |  |  |
| Operation time≥335 min | <0.001 | 1.470 (1.170-1.830) |  |  |
| Intraoperative blood loss≥200 ml | 0.019 | 1.350 (1.050-1.740) |  |  |
| Blood transfusion | 0.601 | 1.070 (0.829-1.380) |  |  |
| Pretreatment chemotherapy | 0.478 | 1.080 (0.867-1.360) |  |  |
| Postopertive chemotherapy | 0.396 | 0.905 (0.717-1.140) |  |  |
| Complication | 0.022 | 1.290 (1.040-1.610) |  |  |
| CLR≥3.06 | 0.006 | 1.380 (1.100-1.730) | 0.005 | 1.400 (1.110-1.780) |
| NLR≥1.86 | 0.789 | 1.030 (0.826-1.290) |  |  |

**Table S3. Prognostic factors for OS in CRLM patients**

| **Factor** | **Univariate analysis** | | **Multivariate analysis** | |
| --- | --- | --- | --- | --- |
|  | ***P*** | **HR (95%CI)** | ***P*** | **HR (95%CI)** |
| Age≥60 years | 0.599 | 1.090 (0.797-1.480) |  |  |
| Male | 0.608 | 0.920 (0.669-1.270) |  |  |
| BMI≥24kg/m^2^ | 0.997 | 1.000 (0.734-1.360) |  |  |
| Comorbidity | <0.001 | 0.984 (0.719-1.350) |  |  |
| ASA score 3-4 | <0.001 | 0.857 (0.524-1.400) |  |  |
| CEA≥200 ng/ml | 0.641 | 1.180 (0.582-2.410) |  |  |
| Primary site in colon | <0.001 | 1.000 (0.735-1.370) |  |  |
| Right hemicolon | <0.001 | 1.050 (0.714-1.550) |  |  |
| Diameter of liver metastases≥5 cm | <0.001 | 1.180 (0.716-1.960) |  |  |
| Multiple liver metastases | 0.001 | 1.740 (1.250-2.410) |  |  |
| Bilobar liver distribution | <0.001 | 1.610 (1.170-2.200) |  |  |
| Poor differentiation | <0.001 | 1.190 (0.847-1.680) |  |  |
| T3-T4 stage | 0.011 | 3.160 (1.300-7.700) | 0.008 | 3.440 (1.390-8.560) |
| Positive lymph node metastasis | <0.001 | 2.680 (1.740-4.140) | <0.001 | 2.720 (1.740-4.240) |
| Extrahepatic metastases | <0.001 | 1.290 (0.778-2.130) |  |  |
| Concomitant RFA | <0.001 | 2.160 (1.440-3.230) |  |  |
| R0 resection | 0.001 | 0.578 (0.416-0.803) |  |  |
| Major liver resection | <0.001 | 2.030 (1.480-2.790) | 0.001 | 2.190 (1.370-3.500) |
| Hepatic portal occlusion | 0.045 | 1.410 (1.010-1.970) | 0.029 | 0.595 (0.374-0.948) |
| All laparoscopic operation | 0.006 | 0.505 (0.309-0.826) |  |  |
| Operation time≥335 min | <0.001 | 1.860 (1.350-2.550) | 0.006 | 1.710 (1.170-2.500) |
| Intraoperative blood loss≥200 ml | 0.063 | 1.390 (0.983-1.970) |  |  |
| Blood transfusion | 0.038 | 1.440 (1.020-2.030) |  |  |
| Pretreatment chemotherapy | 0.018 | 1.470 (1.070-2.030) |  |  |
| Postopertive chemotherapy | <0.001 | 0.501 (0.366-0.686) | <0.001 | 0.431 (0.310-0.599) |
| Complication | 0.0155 | 1.470 (1.080-2.010) |  |  |
| CLR≥3.06 | 0.003 | 1.630 (1.190-2.230) | 0.003 | 1.660 (1.180-2.320) |
| NLR≥1.86 | 0.666 | 1.070 (0.785-1.460) |  |  |
