# Supplementary material for: An elevated preoperative cholesterol-to-lymphocyte ratio predicts unfavourable outcomes in colorectal cancer liver metastasis patients receiving simultaneous resections: a retrospective study
Source: BMC Surg. 2023 May 16;23:131. doi: 10.1186/s12893-023-01988-7 (PMC10190004; doi:10.1186/s12893-023-01988-7)
Supplement: Supplementary file 3 — Additional File 3: Supplementary Figures [file 12893_2023_1988_MOESM3_ESM.docx]

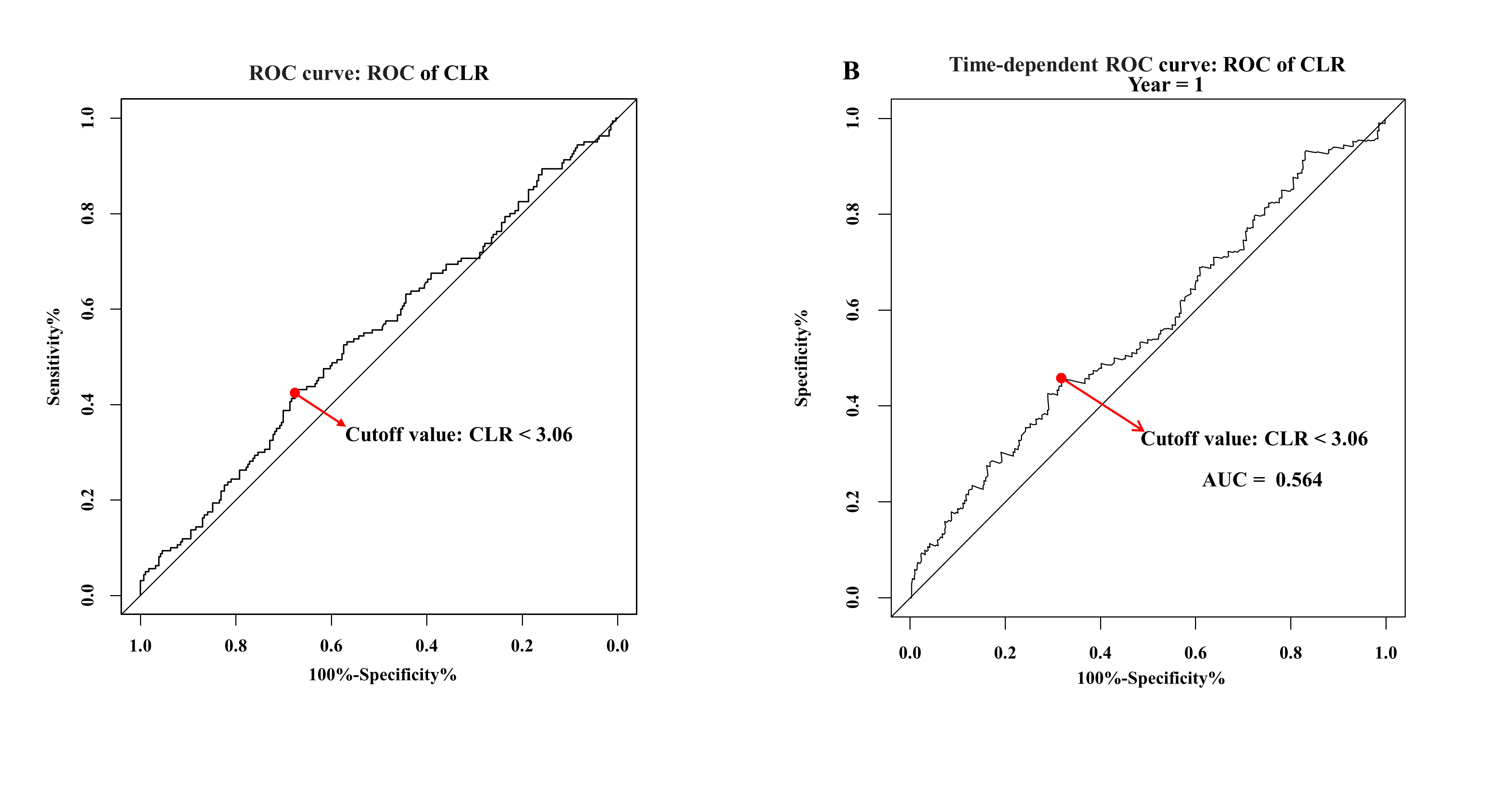


Figure S1 Receiver operating characteristics (ROC) curve analysis of the preoperative cholesterol-to-lymphocyte ratio (CLR) for the detection of mortality.


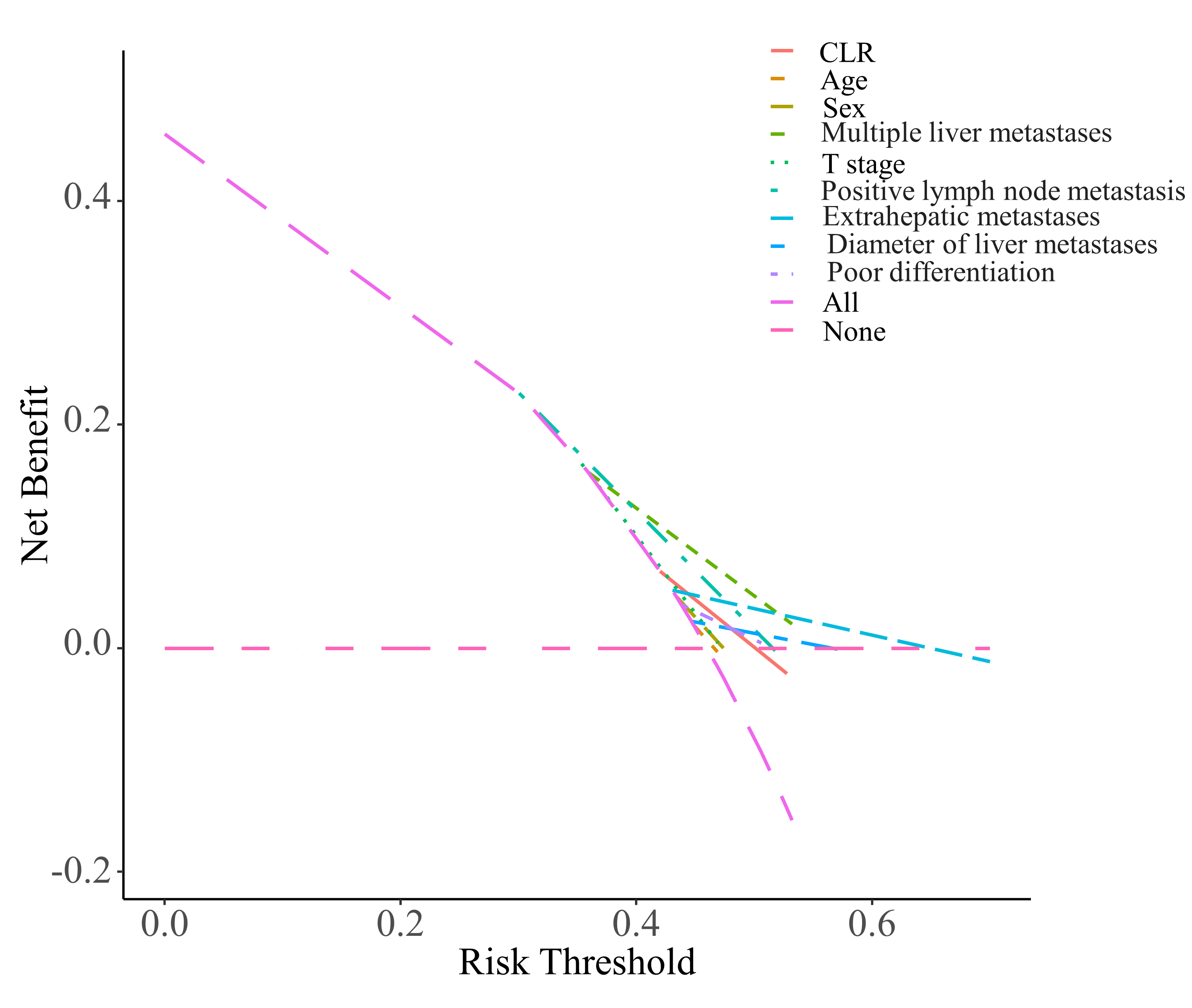


Figure S2 Decision curve analysis (DCA) for the net-benefit of cholesterol-to-lymphocyte ratio (CLR) for progression-free survival (PFS).


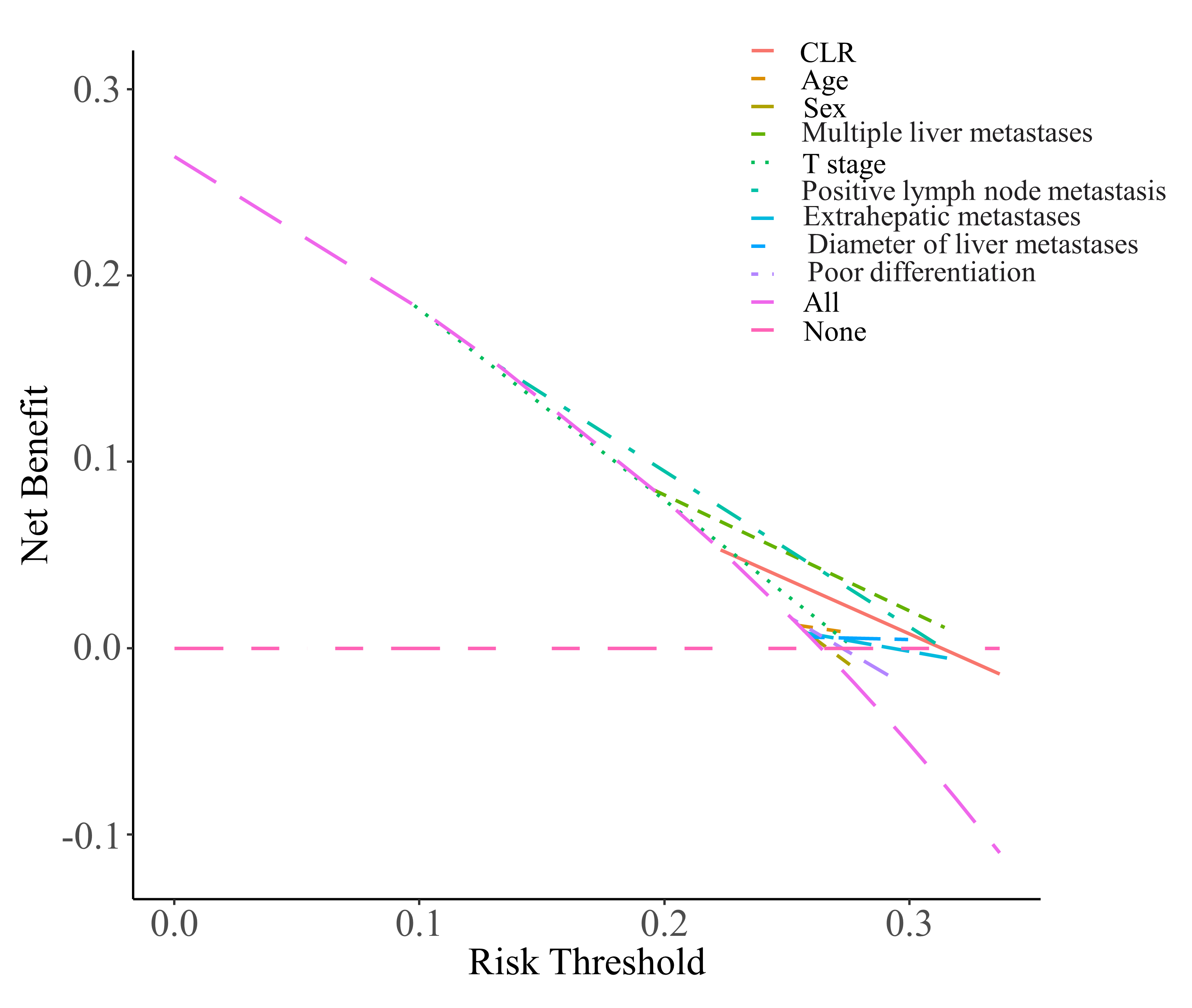


Figure S3 Decision curve analysis (DCA) for the net-benefit of cholesterol-to-lymphocyte ratio (CLR) for overall survival (OS).
